# Facilitating recognition of crowded faces with presaccadic attention

**DOI:** 10.3389/fnhum.2014.00103

**Published:** 2014-02-28

**Authors:** Benjamin A. Wolfe, David Whitney

**Affiliations:** Department of Psychology, University of CaliforniaBerkeley, CA, USA

**Keywords:** crowding, saccades, presaccadic information, presaccadic attention, visually guided action

## Abstract

In daily life, we make several saccades per second to objects we cannot normally recognize in the periphery due to visual crowding. While we are aware of the presence of these objects, we cannot identify them and may, at best, only know that an object is present at a particular location. The process of planning a saccade involves a presaccadic attentional component known to be critical for saccadic accuracy, but whether this or other presaccadic processes facilitate object identification as opposed to object detection—especially with high level natural objects like faces—is less clear. In the following experiments, we show that presaccadic information about a crowded face reduces the deleterious effect of crowding, facilitating discrimination of two emotional faces, even when the target face is never foveated. While accurate identification of crowded objects is possible in the absence of a saccade, accurate identification of a crowded object is considerably facilitated by presaccadic attention. Our results provide converging evidence for a selective increase in available information about high level objects, such as faces, at a presaccadic stage.

## Introduction

Observers make several saccades per second to foveate objects in the world, since objects near other objects are often crowded from our awareness. We can see these objects and we have a sense of where they are in visual space, but we cannot identify them without saccading to and foveating them. This is the underlying assumption of much of our visual system—that, in order to better identify an object, we must make a saccade to it—and until recently, the focus had been on how do we make a saccade, rather than what information do we acquire in the process. Saccadic eye movements have been studied for over a century (beginning with Javal, 1878, who coined the term; translated by Huey, 1908; see Kowler, 2011) for an extensive review of the current state of the art. However, it is only relatively recently, that we have started to ask about what information is acquired prior to the start of a saccade. In particular, we make saccades to crowded objects in order to identify them.

Natural scenes are rich with objects to the point of being cluttered, and this results in visual crowding, a central problem of conscious vision. Visual crowding is the inability to identify an object in the periphery when it is surrounded by other stimuli (Bouma, 1970) and can be operationally defined as the change in the ability to identify an object in the periphery as a result of proximate objects (flankers) in space or time, often phenomenologically reported as a “jumbled” percept of the object and its proximate flankers. Crowding is not a problem of detection; an object or feature is perceived to be present at a location, but it is unidentifiable and its features are jumbled (see Korte, 1923 as translated in Pelli et al., 2004; Strasburger et al., 2011). Crowding has been studied extensively (reviews in Levi, 2008; Pelli, 2008; Whitney and Levi, 2011) with the vast majority of this research having been performed with comparatively simple combinations of features, or letters (Chung et al., 2007; Balas et al., 2009; Grainger et al., 2010; Jeon et al., 2010; Schotter et al., 2012). However, crowding also occurs selectively between objects (Wallace and Tjan, 2011) and faces (Louie et al., 2007; Farzin et al., 2009; Fischer and Whitney, 2011). In addition, considerable research has been done on the impact of flanker configuration on the strength and reduction of crowding (Livne and Sagi, 2007, 2010; Malania et al., 2007; Sayim et al., 2008, 2010; Chakravarthi and Pelli, 2011; Manassi et al., 2012). Crowding often ceases to be a problem once the crowded object has been foveated (Pelli and Tillman, 2008), yet the perceptual consequences of information acquired prior to the saccade on crowding have barely been investigated.

It is therefore reasonable that saccade planning might reduce crowding. Making a saccade to an uncrowded target has been shown to facilitate identification (Remington, 1980) and more recent work has shown that presaccadic attention is key to both the saccade planning process and post-saccade identification of the target object (Kowler et al., 1995; Schneider and Deubel, 1995). However, these studies of presaccadic attention have been limited to relatively simple stimuli (e.g., the “E” and “mirror-E” stimuli used in Schneider and Deubel (1995) and Deubel and Schneider (1996). While presaccadic attention is undoubtedly an important mechanism for presaccadic information acquisition, recent work by Wurtz (2008) and Wurtz et al. (2011), building on the extensive corollary discharge literature, has suggested that the corollary discharge prior to a saccade may act as a trigger for non-attentional facilitation in the same time window. This facilitation would be complementary to presaccadic attention as described by Schneider, Deubel and Kowler respectively, and suggests that there may be alternate mechanisms by which the visual system acquires detailed information about a saccade target prior to a saccade.

Certainly, making a saccade to a crowded object and foveating that object thereafter breaks crowding and allows for the crowded object to be easily identified (Pelli and Tillman, 2008)—but, given the results of recent studies (Kowler et al., 1995; Schneider and Deubel, 1995; Deubel and Schneider, 1996) examining presaccadic attention, we wondered if presaccadically acquired information could reduce crowding on its own. Several recent studies have suggested that saccadic eye movements are accompanied by enhanced attentional resolution (Deubel, 1995; Deubel and Schneider, 1996; Baldauf et al., 2006), and reduced critical spacing in crowding of features or letters (Harrison et al., 2013a).

As we have discussed, crowding has a well-known adverse impact on identification (as reviewed by Pelli et al., 2004; Pelli, 2008; Whitney and Levi, 2011), but it does not impact saccadic accuracy to crowded targets (Vlaskamp and Hooge, 2006), suggesting that sufficient position information is available for accurate saccade planning to crowded targets. More recently, Harrison et al., 2013a) showed that saccades to crowded Gabors acted to reduce the effects of crowding, with the orientation of the Gabors becoming more identifiable by subjects when the Gabors were presented in the 50 ms immediately prior to saccade onset. While Harrison et al. found a reduction in crowding from presaccadic input alone, their results, while suggestive, do not tell us if the same effect might be found with crowded faces. Additional striking work by Harrison et al. (2013b), using a set of letters as stimuli, has shown that visual features can be presaccadically remapped to induce crowding, indicating that saccadic remapping precedes at least one stage of crowding. Incidentally, their work also confirmed the earlier results of Vlaskamp and Hooge with different stimuli, showing that crowding does not impact saccade accuracy.

While earlier work (Kowler et al., 1995; Deubel and Schneider, 1996) demonstrated the crucial role presaccadic attention plays in the saccade planning process, they did not investigate the perceptual consequences of the information that is acquired prior to the saccade. Most importantly for our purposes, the studies cited above on presaccadic attention did not explicitly measure crowding, as noted by Harrison et al. (2013a); c.f., van Koningsbruggen and Buonocore (2013). Moreover, all the work done to date on saccadic amelioration of crowding has used Gabors as stimuli, which, while revealing, are not as complex as stimuli that we commonly saccade to, namely, faces. It remains unclear if the results of Harrison et al. (2013a) extend to more complex and naturalistic face stimuli (Maurer et al., 2002; McKone et al., 2007).

While Harrison et al. (2013a,b) found a modulation of crowding from presaccadic input alone, it remains unclear if there results extend to other visually complex stimuli such as faces. Thus, the experiments of Harrison et al. prompt another question: does face recognition in crowded scenes also benefit from saccades? In the following experiments, we asked if saccades to crowded faces could diminish the effects of crowding, making the faces more identifiable. Gabors and faces are processed at different levels of analysis, faces selectively crowd each other (Louie et al., 2007; Farzin et al., 2009; Haberman and Whitney, 2009), and Gabor (or letter) crowding does not account for the crowding of faces (Whitney and Levi, 2011). Therefore, although saccades might mitigate crowding of features, they may or may not modulate crowding of high-level objects such as faces. In this study, we tested whether presaccadic processes might improve recognition of crowded faces.

## Methods

### Display setup

The experiments were performed using Matlab 2010a (Mathworks; Natick, MA) the Psychtoolbox (Brainard, 1997; Pelli, 1997) and the Eyelink Toolbox (Cornelissen et al., 2002) on a Mac Mini (Apple; Cupertino, CA). Stimuli were displayed on a 47 cm Samsung cathode ray tube display 57 cm from the subject; the display resolution was set to a resolution of 1024 × 768 at 60 Hz in all experiments.

### Subjects

Four subjects (two female; mean age 24) with extensive experience with psychophysical tasks, including the first author (BW), participated in the first experiment. With the exception of BW, all other subjects in the first experiment were naïve to the purpose of the experiment; and had normal or corrected to normal vision. Two subjects (BW and one naïve subject) participated in the control experiment. All subjects provided informed consent as required by the IRB at the University of California, Berkeley in accordance with the Declaration of Helsinki. All subjects received a minimum of 1000 trials of practice prior to any data collection.

### Stimuli

**Figure 1:**
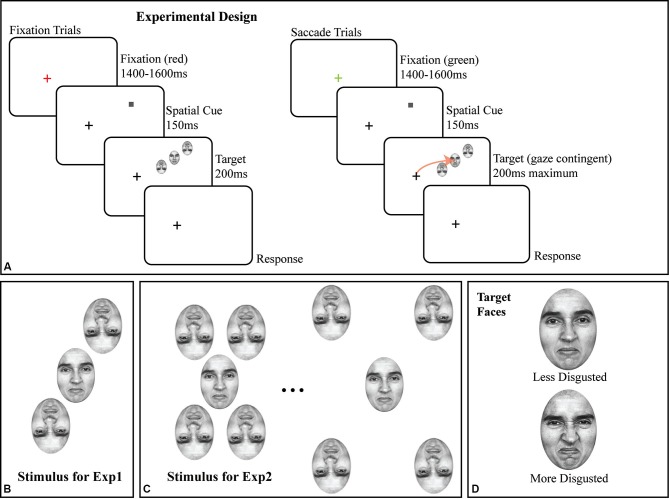
**(A)** Schematic of trial structure in fixation and saccade conditions, with details of the stimuli. Note that the screen-by-screen schematic shows the crowded array in the upper right visual field; in all experiments, the crowded array was randomly presented in the upper and lower right visual field on a trial-by-trial basis. The size of the target face and flankers are exaggerated for illustration; all faces in all experiments were 2 × 3°. The arrow in the saccade trial illustrates the subject’s saccade to the target face. **(B)** Crowded stimulus array used in Experiment 1 with two inverted flanking faces. **(C)** Illustration of crowded stimulus array as used in Experiment 2, showing two of five possible target-flanker spacings with four inverted flanking faces. **(D)** The less disgusted and more disgusted target faces used in both experiments.

All stimuli were faces originally from Ekman’s Pictures of Facial Affect (POFA as used by Fischer and Whitney, 2011); subjects were trained on identifying two emotional faces (target stimuli) prior to any data collection (Figure 1D). All faces were portraits of a single Caucasian female, initially morphed between a neutral and a disgusted expression across 48 intermediate, computer-generated faces, corrected to have the same mean luminance. All experiments used a subset of this space, from a moderately disgusted face (#25; halfway through the morph space) to a fully disgusted face (#50; see Figure 1D for both). All flanking stimuli were randomly sampled around the median point (e.g., halfway between “moderately disgusted” and “disgusted”) of the two target faces. In all experiments, flankers were inverted (as shown in Figures 1B, C) to avoid subject confusion in saccade targeting as well as to reduce possible flanker substitutions and perceptual pooling (Fischer and Whitney, 2011), and to reduce incorrectly directed saccades. Given a constant flanker-target spacing, flanker inversion reduces crowding, but does not eliminate it (Louie et al., 2007); by reducing the target-flanker spacing (Figure 1), we maximized crowding.

In all experiments, the target face and flankers were presented in the right visual field, 10° from the fixation cross, with the flankers in a radial orientation (Figures 1B, C) around the upright target face. Each face subtended a 3 × 2° region on the display. Note that in Figure 1A, the crowded faces are shown in the upper right quadrant of the screen; the presentation location was randomized between the upper right and lower right quadrants on a trial by trial basis. In Experiment 2 (Figure 1C), we added two additional inverted flankers to the array to maximize task difficulty, one above and to the left of the target face and a second below and to the right of the target face to better assess the impact of inverted faces as flankers. Target-flanker spacing (center to center) remained constant at 3° for all trials in Experiment 1 and was varied on a trial-by-trial basis in Experiment 2. Experiment 2 is solely a control for the presence of crowding with face stimuli, since target-flanker spacing remained constant at 3° in Experiment 1. In Experiment 2, target-flanker spacing varied on a trial-by-trial basis from 2.5° to 4.5° in the saccade trials and 3–5° in the fixation trials in steps of 0.5°.

### Trial sequence

Each trial, in all two experiments, began with a red or green fixation cross for 1400–1500 ms (randomized) on a light gray background (39.68 cd/m^2^). The fixation cross was onscreen at all times during the trial (Figure 1A). All stimuli were presented gaze-contingently with eye position monitored throughout the trial. If, at the start of the trial, the fixation cross was green, it signaled a saccade trial, red signaled a fixation trial. Subjects were then shown a valid spatial cue (a 0.25° dark gray square) for 150 ms at the location where the target face would appear. Once the cue appeared, the fixation cross became dark gray and remained so for the remainder of the trial. The cue was then removed from the screen and was immediately followed by the target face and its inverted flankers (Figures 1B, C for detail) centered on the location where the cue had appeared for a maximum of 200 ms. The location of the crowded stimulus array (the target face and the two flankers) was jittered by up to ±1° of visual angle in the *X* and *Y* dimensions on a trial-by-trial basis to avoid stereotyped saccades. In the fixation condition, the stimulus was onscreen for the full 200 ms. In the saccade condition, the stimulus was onscreen for 200 ms or until the saccade was initiated, whichever came first. Eye position was monitored online at 1000 Hz (see Section Eyetracking), and in all saccade trials, any deviation of the subject’s point of gaze greater than 0.5° was treated as the start of a saccade and resulted in the target face being immediately removed from the display. As a result of this conservative ocular motion threshold, the faces remained onscreen for 70 ms, on average, during the saccade trials and reflects a certain degree of saccade planning during the cue period. Gaze-contingent control was maintained throughout the experiment; any deviation from fixation in fixation trials greater than 0.5° initiated the removal of the stimulus, and the trial being redone at the end of the block.

Once the stimulus was removed, subjects made two responses using the keyboard; first, a two-Alternative Forced Choice (2AFC) identification response (was the target presented face A [less disgusted] or face B [more disgusted], distinguishing between the two faces shown in Figure 1D) and second, a 5AFC confidence rating, with a rating of 1 indicating a total lack of confidence in their identification response and a rating of 5 indicating total confidence in their identification response on that trial. The second keyboard response triggered the next trial after a 2 s pause to allow subjects to move their eyes back to the start location (fixation cross). Collecting this additional 5AFC response wherein subjects rated their confidence in their own responses allowed us to better probe the effect of the saccade; subjects reported that they felt more confident in their own responses when making a saccade than when fixating.

### Block sequence

In Experiment 1, subjects participated in 5 runs of 216 trials each; each run consisted of eight 27-trial blocks which alternated between the saccade or fixation conditions. In Experiment 2, subjects participated in 10 runs of 240 trials each, divided into eight 30-trial blocks which alternated between the saccade and fixation conditions.

### Eyetracking

Eyetracking was performed using an Eyelink 1000 (SR Research; Mississauga, ON, Canada) with a level desktop camera; data was recorded monocularly (right eye for all subjects) at 1000 Hz and saccade analysis was performed offline using the Eyelink parser. A saccade was defined as the first time point at which the velocity exceeded 30°/s and the acceleration exceeded 8000°/s^2^. In addition, a motion threshold was used to delay the start of each saccade until the eye had moved at least 0.15°. All subjects were stabilized on a chinrest during all experiments. Subjects were calibrated using a standard 9-point grid.

Subjects’ eye movements were recorded at all times during the experiment, and eye movements were only permitted in the saccade blocks during a specified response window after the crowded face was presented. Rather than attempt to parse saccades in real time, which would have introduced an unacceptable delay, raw gaze position was continually monitored over the realtime link between the eyetracking computer and the stimulus computer, and any deviation in eye position greater than 0.5° in any direction was treated as the beginning of a saccade. In the saccade trials, any deviation in excess of 0.5° resulted in the stimuli being immediately removed from the screen to prevent inadvertent foveation of the stimuli. Subjects were required to maintain accurate fixation during fixation trials, and we used the same criteria as in saccade trials to prevent inadvertent examination of the target faces. Eye movements at any other time, or during the fixation blocks, resulted in the trial being aborted, the screen going red for 2 s and the subject repeating the trial in random order at the end of the block.

### Analysis

Behavioral and eyetracking data were analyzed offline using custom Matlab scripts and S-R Research’s EDFMEX tool. All trials in the saccade blocks were filtered by landing location; subjects were required to land their first saccade within ±1.5° of the center of the upright target face, and were otherwise discarded. Fixation trials were automatically discarded and rerun, as described in the previous section, if the subject’s eye moved more than 0.5° at any time during a fixation trial. All data was collapsed across the upper and lower visual field presentation locations.

The confidence ratings and responses for each trial were used to calculate two Receiver Operating Characteristic (ROC) curves, one for the fixation condition and one for the saccade condition. The data points used to plot each ROC curve were calculated using the following procedure from Murdock (1965). Within each eye movement condition (saccade or fixation), we sorted each trial based on which face was presented (the less disgusted or more disgusted face) and then further sorted them based on the accuracy of subject’s 2AFC identification responses and their 5AFC confidence ratings. The combination of the subject’s 2AFC response and the 5-point rating of their confidence in their response together gave us a measure on each trial of how confident the subject was on a scale from 1–10 that they were presented with a more disgusted face. For example, if a subject responded that they were shown a “less disgusted” face and were highly confident in their response (i.e., gave a rating of 5), that meant that they were certain that they did not see a “more disgusted” face. In other words, the subject would be unconfident that they saw a more disgusted face, and this would be assigned a rating of 1 on the 1–10 scale. On the other hand, if the subject responded that they were shown a more disgusted face and were confident in their response (i.e., gave a rating of 5), they were confident that they saw a disgusted face, and this response would be assigned a rating of 10 on the 1–10 scale. Doing this across our entire set of trials within a given eye movement condition allowed us to reclassify our original 2AFC and 5AFC responses on a 10-point scale for each stimulus condition. We then calculated the number of responses for each rating (1 through 10) within each stimulus condition (disgusted or less disgusted) separately. We then calculated the proportion of the total number of trials within each stimulus condition that fell into each bin.

Using these proportions, we generated a set of cumulative conditional probabilities for each stimulus condition. In essence, for a given rating on our 10-point scale, we asked what proportion of responses would be encompassed by a criterion (for correctly detecting a disgusted face) set at a given point on the scale or greater. So, the cumulative conditional probability for a rating of 1 on this scale encompasses all of our transformed responses, while the cumulative conditional probability for a rating of 5 is considerably less than that, reflecting the more stringent criterion. In each case, we can calculate hits (the cumulative probability for a given criterion when the subject was shown the disgusted face and correctly identified it) and false alarms (the cumulative probability when the subject was shown the less disgusted face and misidentified it as the disgusted face). The entire set of hit rates and false alarm rates for each criterion form the points of our ROC curve. We then calculated the area under curve (AUC) using the trapezoidal integration function in Matlab. Significance was tested with *Z*-tests, the *Z*-scores were converted to *p*-values using the standard normal distribution on the AUC (Hanley and McNeil, 1982). Standard error for AUC was estimated using the method of Hanley and McNeil (1983).

## Results

### Experiment 1

Experiment 1 tested whether presaccadic information facilitated identification of a crowded emotional face in the absence of foveating the face; as described in Section Methods, target-flanker spacing was held constant at 3° throughout the experiment. The data were analyzed using a ±1.5° window centered on the target face for saccade trials, ensuring that the saccades in question were well-localized to the crowded face and that the saccade on a given trial had not landed at the location where an inverted flanker had been present prior to the saccade.

**Figure 2:**
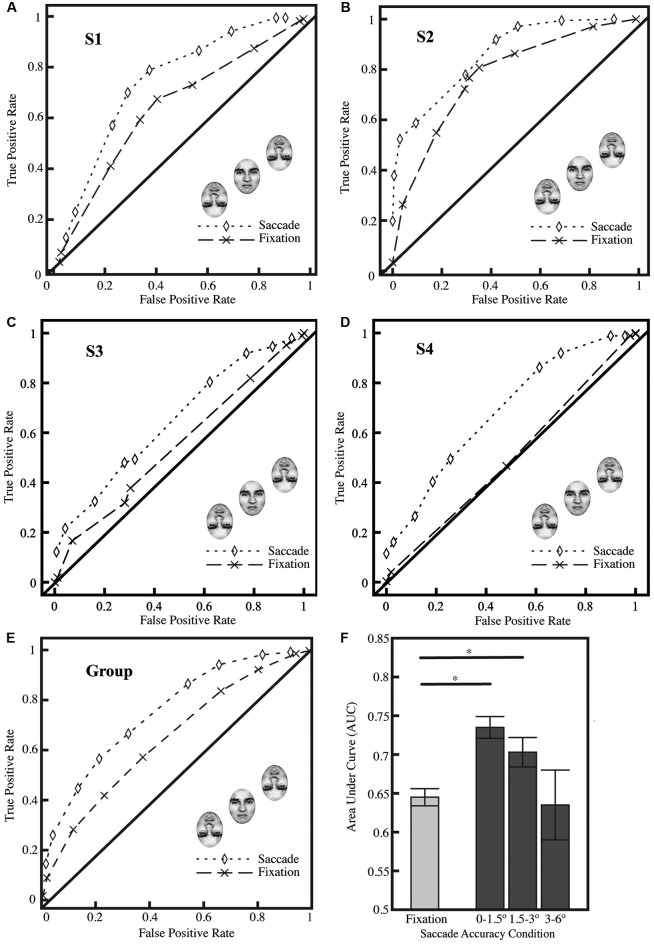
**Results from Experiment 1. (A–D)** ROC curves from all four subjects showing performance in fixation and saccade conditions (significantly different; *p* < 0.01; all 4 subjects show significant differences individually) with saccade landings restricted to ±1.5° from the center of the target face. Three of the four subjects **(A–C)** show significant performance in the fixation condition (comparison vs. AUC of 0.5; *p* < 0.01). **(E)** ROC curves for all subjects (*n* = 4) collapsed; saccade landings restricted as in **A–D**. **(F)** Average area under curve (AUC) for fixation (**F**; left bar) and saccade conditions (**F**; right). Trials on which the saccades landed up to ±1.5° from the center of the face showed the greatest improvement in performance; the effect was reduced with less accurate saccades (landing ±1.5–3.0° from the center of the face). The AUC in the fixation represents a baseline level of performance on the task. A *Z*-test was performed and the *Z*-score converted to a *p*-value using the standard normal distribution (Hanley and McNeil, 1982), and was then used to compare AUCs between the fixation condition and difference saccade accuracy windows; asterisks indicate significance at *p* < 0.05. Standard errors were estimated using the procedure of Hanley and McNeil (1983).

As a group (Figure 2E) and as individuals (Figures 2A–D), subjects found the identification task challenging, but they were able to perform the identification task above chance in the fixation condition (group AUC, 0.645 for fixation; *p* < 0.0001; two-tailed *Z*-test converted to *p*-value; per the procedure of Hanley and McNeil, 1982), and subjects achieved significantly better performance in the saccade condition (group AUC, 0.735, *p* < 0.0001); the AUC for the saccade and fixation conditions were significantly different (*p* < 0.0001). Three out of four subjects, individually, were significantly above chance in the fixation condition (*p* < 0.01), indicating their basic competence at the task, and all subjects individually showed significant differences between the saccade and fixation conditions (Figures 2A–D; all *p* < 0.01). We performed additional analyses (Figure 2F) excluding the central region previously analyzed (1.5°) and found similar results for the region of 1.5–3° from the center of the crowded face (saccade AUC, 0.703, *p* < 0.0001); grossly inaccurate landings (greater than 3°) did not show any significant difference between saccade and fixation performance at the group level. On average, 51% of saccades landed within ±1.5° of the center of the upright target face; an additional 42% landed within ±1.5–3°.

### Experiment 2

To validate our procedure in Experiment 1, we performed a control experiment with two subjects to verify that subjects’ reduction in performance in the fixation task was due to crowding rather than to other factors, such as limits on visual acuity. To do this we systematically varied the target-flanker spacing in Experiment 2. Observing a decrease in performance with a decrease in target-flanker spacing, and an increase in performance with an increase in target-flanker spacing would indicate that crowding did, in fact, occur with the stimulus arrangement we used in Experiment 1. In Experiment 2, we added two additional inverted flankers (Figure 1C) and used two offset ranges of target-flanker spacings, from 2.5° to 4.5° in the saccade trials, and 3–5° in the fixation trials, as well as adding a baseline no-flanker stimulus in both conditions as a point of comparison.

**Figure 3:**
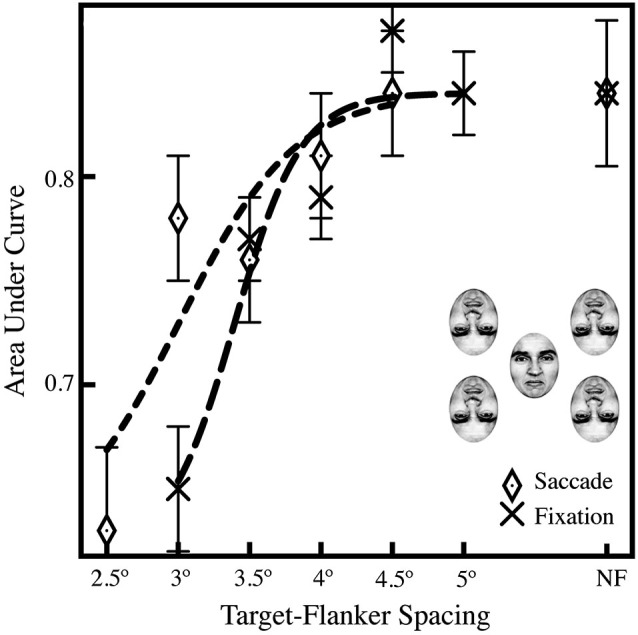
**Averaged AUCs for Experiment 2, showing performance across a range of target-flanker spacings (2.5–4.5˚ in the saccade condition; 3–4.5˚ in the fixation condition) and a leftward shift in the saccade condition relative to the fixation baseline, suggesting that the saccade itself may diminish crowding**. The data were fit to a logistic curve using the least-squares method in Matlab. Data in the no-flanker condition (NF, far right) is included as a baseline for comparison. Experiment 2 was performed with four flankers, as shown above the legend, to maximize the effects of crowding. A *Z*-test was performed and the *Z*-score converted to a *p*-value using the standard normal distribution (Hanley and McNeil, 1982), and was then used to compare AUCs between the fixation condition and difference saccade accuracy windows; general significance described in the text (Section Experiment 2). Standard errors were estimated using the procedure of Hanley and McNeil (1983).

In Experiment 2, we found that subjects showed equal performance with spacings in excess of 4° when making saccades and 4.5° when fixating; the average AUCs in Figure 3 show a decrease in performance with a decrease in target-flanker spacing, indicating that crowding impeded identification of the target face. A series of pairwise comparisons across the saccade and fixation conditions showed a significant difference in performance in the 3° target-flanker spacing condition (*p* < 0.001; Bonferroni-corrected α = 0.01); comparisons in the 3.5°, 4°, and 4.5° and no-flanker conditions were all not significant (*p* > 0.01). We do find a significant overall effect of target-flanker spacing on performance; when we compared the closest spacing in the fixation condition (3°) to the farthest (5°), we found a significant difference in performance (*p* < 0.001). In addition, when we compared the closest spacing (3°) to the no-flanker condition, that difference was also significant (*p* < 0.001), which replicates our result in Experiment 1. Critically, subject performance in the no-flanker condition (NF) was identical across the saccade and fixation conditions, indicating that the target face alone was easily recognizable. In addition, we observed that 43% of saccades to crowded faces landed within ±1.5° of the center of the upright target face; an additional 51% landed within ±1.5–3°. This is nearly identical to saccades to unflanked faces where we observed 45% of saccades landed within ±1.5° of the center of the upright target face; an additional 47% landed within ±1.5–3°.

## Discussion

### Experiment 1

This experiment provides the first evidence that executing a saccade to a crowded face improves recognition of that face. Notably, the improvement in identification performance that we report does not arise from direct foveation of the target face. The target face and its flankers were removed from the screen as soon as the subject’s eye deviated from fixation by more than 0.5°, which prevented direct foveation of the face and, incidentally, resulted in subjects having considerably less time to acquire information about the face than in the fixation condition. In the saccade condition, the target face and its flankers were onscreen for an average of 70 ms, and yet subjects could still identify the face they were saccading to more accurately than when they maintained fixation on the cross and identified the face from peripheral information alone.

Although the results of Experiment 1 are intriguing, they do not necessarily indicate a reduction in crowding. The improvement in identification accuracy we observed in this experiment with saccades could be attributed to presaccadic attention alone, a result which would accord with previous findings (Kowler et al., 1995; Schneider and Deubel, 1995; Deubel and Schneider, 1996) although we should distinguish between presaccadic attention and simple covert attention in the absence of a saccade (see additional commentary in Sections Experiment 2 and General Discussion). To determine whether our procedure in Experiment 1 induced crowding and to probe the effect further, we performed a control experiment wherein we manipulated target-flanker spacing across both the saccade and fixation conditions (Experiment 2).

### Experiment 2

In Experiment 2, we found that manipulating the target-flanker spacing as a diagnostic test for the presence of visual crowding resulted in decreased performance with smaller target-flanker spacings; this finding suggests that the stimuli used in Experiment 1 were made less identifiable by crowding and that we may have observed a partial release of crowding with saccades in that experiment. Our results in Experiment 2 show a pattern of decreasing accuracy with decreased target-flanker spacing, but crucially, we find a stable level of performance at larger target-flanker spacing across conditions, and we find an identical level of performance without flankers (Figure 3). Notably, the results in the saccade condition of Experiment 2 hint that there may be a partial saccade-mediated release of crowding with a saccade to a crowded target. We found a significant difference in performance between the saccade and fixation conditions at only one target-flanker spacing (3°) suggesting that a saccade to a crowded face does reduce the effects of crowding, but only in a limited range. We also found significant differences between the closest and furthest spacings in the fixation condition (2.5° and 4.5°) as well as between the closest spacing and the no-flanker condition, indicating that the inverted faces were effective in inducing crowding in our experiments. Given that performance in the no-flanker condition was identical across the saccade and fixation conditions, our findings do not support a purely attentional explanation of the primary effect, as we would expect to see an increase in overall performance with saccades, even with minimal or nonexistent crowding, which does not appear in our data. In addition, the significant difference in performance between the saccade and fixation conditions with 3° target-flanker spacing allows us to distinguish the effects of covert attention, as observed in the fixation condition, from those of presaccadic attention and other presaccadic processes. If the two forms of covert attention were identical, we would expect identical performance at all target-flanker spacings, as well as in the absence of flankers.

### General discussion

Our results suggest that not only does a saccade to a crowded face improve identification performance of isolated target objects, but that presaccadic attention and/or potentially other presaccadic processes triggered by the corollary discharge, as discussed by Harrison et al. (2013a), can provide sufficiently detailed information to discriminate between two faces. If discriminating two emotional faces requires a configural or holistic process (as suggested by Maurer et al., 2002), then our results suggest that configural or holistic info can get through crowding in virtue of presaccadic processes including, perhaps, presaccadic attention. Our results extend prior work that was conducted with simple letter stimuli (Kowler and Blaser, 1995; Schneider and Deubel, 1995; Deubel and Schneider, 1996), rather than the faces we used. Presaccadic attention has been previously shown to be crucial for accurate saccade planning (Kowler and Blaser, 1995) and to improve identification performance with letter stimuli (Deubel and Schneider, 1996), but our work is the first to show that high-level, identifying information, such as is used to discriminate two faces, might be acquired prior to the saccade.

We believe that our results are consistent with those of Harrison et al. (2013a), who found similar results with Gabor stimuli. Particularly, Harrison et al. found that saccadic amelioration of crowding was limited to close target-flanker spacings, and that saccades did not improve identification of weakly crowded targets. Our results suggest that crowded information about features or high-level objects in the visual periphery is, under some circumstances, available to the perceptual system when a saccade is prepared. This information may play a role in presaccadic identification of a saccade target. Acquiring detailed identifying information about a saccade target prior to the saccade itself may have significant implications for our understanding of visual stability (Melcher and Colby, 2008), and, more simply, for how we are able to readily identify objects in the world around us, particularly across eye movements.

The idea that saccade planning, including presaccadic attention, may capture more complex information is not without some precedent (Remington, 1980). For that matter, other work on visual crowding has shown that information about crowded stimuli is blocked from conscious access, but not discarded entirely (Dakin, 2001; Parkes et al., 2001; Fischer and Whitney, 2011). Our results suggest that presaccadic attention can make this information accessible in a way that partially bypasses or releases visual crowding. The information that is rendered inaccessible by visual crowding does not seem to be usefully retrievable in the absence of a saccade. Certainly, covert visual attention as used by our subjects in the fixation condition, is insufficient to relieve crowding to the degree we observe with saccades. However, presaccadic attention is not the only mechanism which might drive our effect. Other presaccadic processes, such as perisaccadic unmasking, as suggested by van Koningsbruggen and Buonocore (2013) in their discussion of the work of Harrison et al. (2013a), may be involved as well. We do not believe that perisaccadic unmasking can explain our effect; unlike the stimuli of Harrison et al., our stimuli were not masked, and were merely removed upon saccade onset. While we believe that our results suggest a distinction might be made between covert attention, as used in the fixation condition, and presaccadic attention, it is entirely possible that our effect is caused by other means, such as the nonattentional presaccadic enhancement suggested by Harrison et al. (2013a).

In summary, these experiments demonstrate a presaccadic enhancement of crowded face recognition, and our results may represent an expansion of the known capabilities of presaccadic attention. Our results demonstrate that presaccadic attention or other presaccadic processes capture sufficient information to discriminate two crowded emotional faces, a task which requires considerably more information than discriminating the orientation of a letter or a Gabor patch. Presaccadic attention may be a privileged form of attention, since simple covert attention as used by our subjects in the fixation condition does not result in the same level of performance, and it appears to be crucial not only for saccade targeting (Kowler and Blaser, 1995), but potentially for identifying the target of the saccade before the eyes are in motion. Saccades have long been known to facilitate perception, but the act of making a saccade seems to enhance perception by acquiring information which is otherwise inaccessible prior to the saccade.

## Author Contributions

Benjamin A. Wolfe and David Whitney designed the experiments; Benjamin A. Wolfe programmed the experiments, collected and analyzed the data, Benjamin A. Wolfe and David Whitney wrote the manuscript.

## Conflict of interest statement

The authors declare that the research was conducted in the absence of any commercial or financial relationships that could be construed as a potential conflict of interest.
